# Ageing differentially affects neural processing of different conflict types—an fMRI study

**DOI:** 10.3389/fnagi.2014.00057

**Published:** 2014-04-07

**Authors:** Margarethe Korsch, Sascha Frühholz, Manfred Herrmann

**Affiliations:** ^1^Department of Neuropsychology and Behavioral Neurobiology, Bremen UniversityBremen, Germany; ^2^Center for Cognitive Sciences (ZKW), Bremen UniversityBremen, Germany; ^3^Swiss Center for Affective Sciences, University of GenevaGeneva, Switzerland; ^4^Neuroscience of Emotions and Affective Dynamics Laboratory, Department of Psychology, University of GenevaGeneva, Switzerland

**Keywords:** ageing, flanker, stimulus-response-conflict, double conflict, stimulus-stimulus-conflict, fMRI

## Abstract

Interference control and conflict resolution is affected by ageing. There is increasing evidence that ageing does not compromise interference control in general but rather shows distinctive effects on different components of interference control. Different conflict types, [e.g., stimulus-stimulus (S-S) or stimulus-response (S-R) conflicts] trigger different cognitive processes and thus activate different neural networks. In the present functional magnetic resonance imaging (fMRI) study, we used a combined Flanker and Stimulus Response Conflict (SRC) task to investigate the effect of ageing on S-S and S-R conflicts. Behavioral data analysis revealed larger SRC effects in elderly. fMRI Results show that both age groups recruited similar regions [caudate nucleus, cingulate gyrus and middle occipital gyrus (MOG)] during Flanker conflict processing. Furthermore, elderly show an additional activation pattern in parietal and frontal areas. In contrast, no common activation of both age groups was found in response to the SRC. These data suggest that ageing has distinctive effects on S-S and S-R conflicts.

## Introduction

Healthy ageing is accompanied by alterations in a wide range of cognitive functions. In addition to behavioral performance, age-related adjustments also become evident with regard to neural correlates of cognitive functions. Several studies have found increased activation in elderly individuals during memory or executive tasks (Cabeza et al., 1997; Grady et al., 1999; Reuter-Lorenz et al., 2000). Based on these data different ageing theories (Cabeza, 2002; Reuter-Lorenz and Cappell, 2008; Park and Reuter-Lorenz, 2009) have emerged to provide a framework for age-specific neural alterations. According to several of these theories increased activation represents a compensatory mechanism that should counteract several physiological alterations of the ageing brain, such as atrophy or alterations in different transmitter system (see Park and Reuter-Lorenz, 2009). According to the “frontal lobe hypothesis” (West, 1996) these alterations are particularly pronounced in the prefrontal cortex (PFC) and also comprise executive control functions such as interference control. Interference control refers to the cognitive ability to extract the task-relevant information and to inhibit task-irrelevant information from simultaneous and competing information streams. In the context of cognitive ageing interference control seems to play a crucial role, since impaired inhibition control or conflict processing compromises other cognitive functions like memory performance (Hasher and Zacks, 1988), and may interact negatively with cognitive changes that usually accompany ageing. Though interference control is widely investigated in young participants, there is still little knowledge about how different components of conflict processing change with advanced age.

Interference control is a mandatory cognitive function for daily-life activities and comprises the ability to regulate and process competing information streams. To investigate the underlying cognitive and neuronal mechanisms of interference control different conflict tasks were introduced, such as the Flanker paradigm (Eriksen and Eriksen, 1974), the Simon paradigm (Simon, 1969), or the Stimulus-Response Conflict (SRC) (Kornblum et al., 1990) paradigm. The Flanker task usually consists of a target stimulus surrounded by two or more distracters, which are congruent or incongruent with the target stimulus. The Simon conflict and the SRC task contain spatial information that can be congruent or incongruent with the expected response side. According to Kornblum et al. (1990) Flanker and SRC conflicts are represented in different stages of stimulus processing and, thus, characterize different types of conflicts. While the Flanker conflict is assumed to originate from the early stage of stimulus encoding (stimulus-stimulus (S-S) conflict), the SRC evolves in the later stage of response selection (S-R conflict). There is converging evidence that SS and SR conflicts rely on distinctive neural networks. A meta-analysis by Nee et al. (2007) shows that S-S conflict processing is associated with an activation of right dorsolateral PFC and the insula whereas S-R conflict resolution recruits parietal regions as well as anterior cingulate and premotor/supplementary motor cortex. Furthermore, various studies introducing different types of conflict processing in the same study group also demonstrate independent neural mechanisms to be engaged in different conflict resolution (e.g., Egner et al., 2007; Fruhholz et al., 2011).

Various neuroimaging studies have proved that elderly participants show increased activation in frontal and parietal areas when faced with incongruent information in conflict tasks (Milham et al., 2002; Langenecker et al., 2004; Lee et al., 2006; Zysset et al., 2007). These findings are in line with cognitive ageing theories stating that elderly employ compensatory neural mechanisms to compensate for an age-related cognitive decline. However, data from several studies challenge the frontal lobe hypothesis and the compensatory hyperactivation hypothesis, and cast doubt on the idea that this theory equally applies for all cognitive functions associated with the PFC. West and Alain (2000) used a Stroop task and demonstrated that the processing of distracters was impaired in elderly, while the processing of the target stimulus remained intact. Importantly, both conflict resolution processes were associated with an EEG signal source in the PFC. Furthermore, a recent meta-analysis (Turner and Spreng, 2012) on inhibition and working memory revealed that age-related differences of neural processing differ depending on the respective cognitive domain. While working memory was characterized by increased activation of dorsolateral PFC (dlPFC), supplementary motor area (SMA) and inferior parietal lobule (IPL) in elderly, age-related differences in inhibition processing became evident in the inferior frontal gyrus (IFG) and the pre-SMA. These findings indicate that cognitive ageing is not associated with a general mechanisms leading to a compensatory increase in PFC activity but rather by a specific modification of the neural networks involved in the processing of a certain task or task component. As this meta-analysis summarizes studies that used different types of conflict tasks (e.g., Flanker task and Simon task) no information is provided about conflict specific ageing effects.

With respect to conflict resolution in elderly subjects there is further evidence for a dissociation of ageing effects on interference control. With regard to behavioral performance, it has been shown that elderly participants show higher congruency effects compared to young participants during the Simon task (van der Lubbe and Verleger, 2002; Kubo-Kawai and Kawai, 2009), while Flanker effects do not differ between young and old participants (Falkenstein et al., 2001; Nieuwenhuis et al., 2002). These data indicate that conflicts in the response selection stage are particularly vulnerable to age-associated changes in inhibition control. Furthermore, Sebastian et al. (2013) reported that age-related neural effects differ in subcomponents of response inhibition as revealed by a hybrid of a Go/Nogo, Simon and a Stop Signal tasks. Thus, ageing does not seem to affect interference control in general, but rather has distinctive effects on different conflict types. However, despite these observations it is difficult to delineate conflict specific neuronal features of ageing, since different conflict types were investigated in independent experimental settings and across independent groups. To the best of our knowledge a recent study from Kawai et al. (2012) was the first to introduce both Flanker and Simon tasks within one experimental design in young and elderly participants using near-infrared spectroscopy (NIRS). Data from this study revealed that during Flanker task processing elderly participants showed increased activation in the right middle frontal gyrus (MFG) and superior frontal gyrus (SFG). In contrast, during the Simon task the elderly group elicited higher activation over bilateral sites corresponding to SFG. These data again demonstrate that an age-related over recruitment in frontal brain areas seems to be dependent on different conflict types. However, the NIRS technique only has a restricted spatial resolution of signal origin, and Kawai et al. (2012) only recorded signal in frontal areas. In the present study we used functional magnetic resonance imaging (fMRI) as a non-invasive method that measures brain activity as a change of blood oxygenation level due to an increase of blood flow. In contrast to the afore-mentioned NIRS technique, fMRI offers a high spatial resolution of neural activity covering the whole brain, and particularly also allows for the investigation of subcortical and deeper brain regions.

The aim of the present fMRI study was to investigate whether age-dependent changes in conflict processing differ according to the type of conflict. Therefore, we introduced a combined Flanker and S-R task which allows for the separate analysis of S-S and S-R conflicts as well as a combination of both within one experimental setup. We hypothesized that (1) elderly show enlarged S-R conflict effects while we expect no increased conflict effects regarding the Flanker task in elderly, and (2) ageing will differentially affect brain activation patterns in Flanker and SRC task processing.

## Materials and methods

### Participants

Nineteen healthy elderly (10 males; mean age 70.26 years, *SD* = 3.49) and 20 healthy young (10 males; mean age 22.95 years, *SD* = 2.72) volunteers participated in the experiment. According to the Edinburgh Handedness Inventory Scale (Oldfield, 1971) all participants were right-handed. No subject reported a history of neurologic or psychiatric disorders. All participants had normal or corrected to normal vision and a comprehensive neuropsychological testing was conducted to exclude participants performing outside age-adjusted norms. All participants gave informed and written consent for their participation and the study was approved by the local ethics committee of the University of Bremen.

### Stimulus material

**Figure 1:**
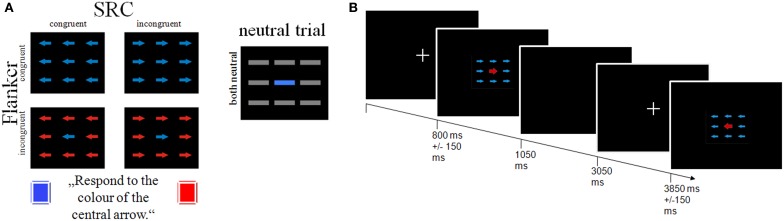
**Schematic illustration of the study design. (A)** The combination of the Flanker and SRC task results in four conditions: a Flanker and SRC congruent condition (FcSc); a Flanker congruent and SRC incongruent condition (FcSi), a Flanker incongruent and SRC congruent condition (FiSc), and a double incongruent condition (FiSi). **(B)** Depiction of trial sequence and timing parameters.

Stimuli consisted of nine colored (blue or red) arrows, arranged in three rows in the center of the screen (see Figure 1). Participants were instructed to attend to the color of the central arrow and to press a response button with their right and left index finger according to target color. Flanker conflict was induced by the arrows surrounding the target with either the same color (congruent Flanker condition) or the color associated with the opposite response side (incongruent Flanker condition). In addition S-R conflict was induced by the orientation of the arrows that was either congruent or incongruent to the response side. The experimental setup resulted in four combinations of the Flanker and SRC conditions: (1) a double congruency condition with corresponding color and direction information (FcSc = Flanker congruent, SRC congruent); a single Flanker/SRC condition with (2) incongruent color and congruent direction information (FiSc) or (3) congruent color and incongruent direction information (FcSi), and (4) a double incongruency condition with incongruent color and incongruent direction information of the stimuli (FiSi). In addition we introduced a neutral condition with horizontal gray bars instead of arrows. Each condition consisted of 72 trials presented in six blocks (each with 60 trials) and separated by short breaks of 10 s. The trial sequence was pseudo-randomized to avoid trial succession effects. Each trial started with a fixation cross presented for 800 ± 150 ms in the center of the screen. The stimulus appeared for 250 ms followed by a blank screen for 2000 ms. The stimulation software Presentation® (Neurobehavioral Systems; https://nbs.neuro-bs.com) was used to display stimuli via a JVC video projector onto a projection screen at the rear end of the fMRI scanner with a viewing distance of about 38 cm.

### Image acquisition

A 3-T SIEMENS Magnetom Allegra System (Siemens, Erlangen, Germany) with a T2^*^-weighted gradient echo-planar imaging (EPI) sequence (28 contiguous slices aligned to AC-PC place, slice thickness 4 mm, no gap, *TR* = 1.5 s, *TE* = 30 ms, *FA* = 73°, in-plane resolution 3 × 3 mm) and a manufacturer supplied circularly polarized head coil signal was used for functional imaging data acquisition.

### Image analysis

Pre-processing and functional data analysis was performed with the Statistical Parametric Mapping software SPM (Version 5; Welcome Department of Cognitive Neurology, London, UK). The first ten volumes of each functional data set were discarded. Scans were re-aligned to the first volume, slice-time corrected and spatially normalized to the standard SPM EPI template in MNI space. Smoothing of the data was conducted using a 8 × 8 × 10 mm full width at half maximum (FWHM) Gaussian kernel. Data were high-pass filtered (128 Hz) to remove low frequency signal drifts. A first-order autoregression model (AR-1) was used to correct temporal autocorrelations.

Regressors were defined by delta functions convolved with a canonical hemodynamic response function (HRF) at each stimulus onset. The design matrix consisted of five regressors representing trials with correct responses (FcSc, FiSc, FcSi, FiSi, neutral), an additional regressor for erroneous trials as well as six motion regressors containing movement parameters obtained during realignment.

For the first-level analysis t-contrasts were calculated for each participant. Each contrast included a single conflict condition compared to the double congruent condition ([Fisc > FcSc]; [FcSi > FcSc]; [Fisi > FcSc]). Group specific activation was determined with a one sample *t*-test for each contrast and each group. To investigate age-dependent differences each contrast was entered into a second level analysis using two sample *t*-tests with the young and elderly participants groups as independent groups. For exploratory purposes we followed an experimental approach with a similar task design established in our lab in previous studies (Fruhholz et al., 2011). Contrasts were thresholded at *p* < 0.001 (uncorrected) combined with a cluster extent threshold of *k* = 9. In addition, group contrasts thresholded at *p* < 0.005 for young and elderly participants respectively were used as an inclusive mask to exclude task irrelevant activation differences. We used the conjunction null hypothesis (Nichols et al., 2005; *p* < 0.005 (uncorrected), *k* = 9) for each contrast to identify common activation patterns.

## Results

### Behavioral data

**Figure 2:**
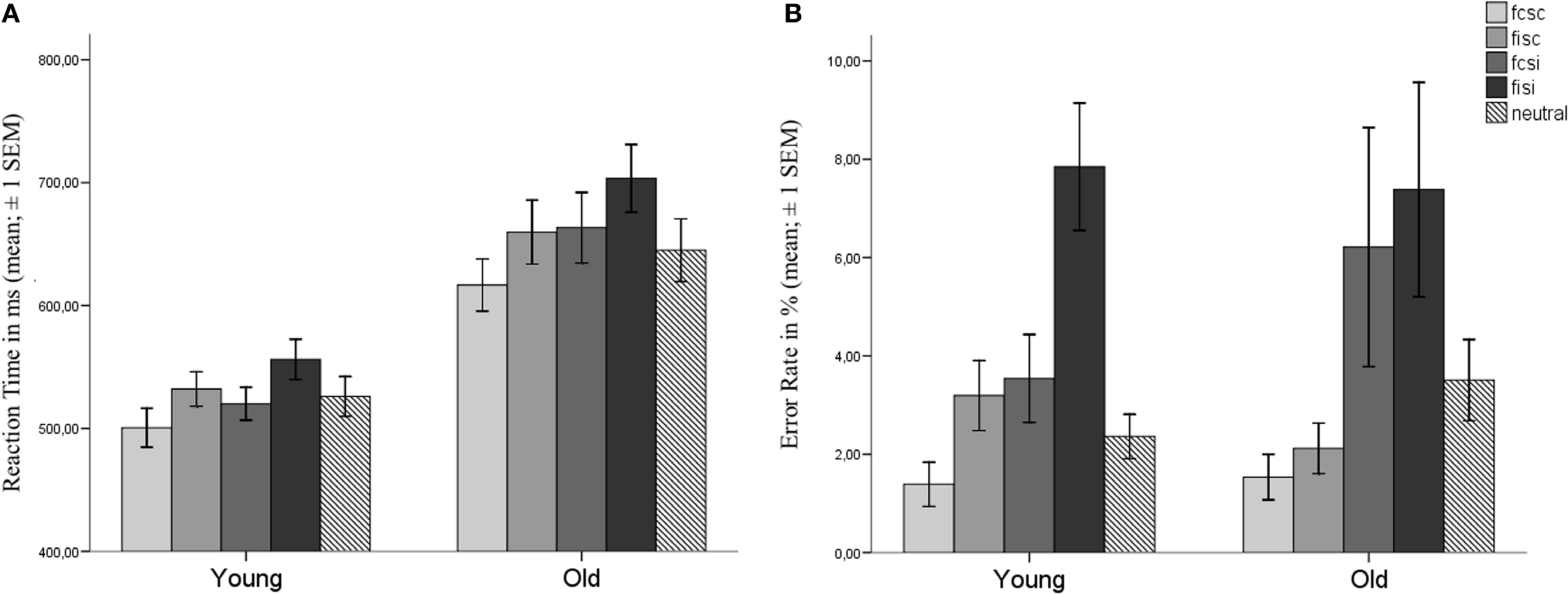
**Behavioral data. (A)** reaction times and **(B)** error rates for young and old participants. Error bars show the standard error of the mean (SEM). Bars represent the different experimental conditions with congruent Flanker and SRC (FcSc) trials, incongruent Flanker and congruent SRC (FiSc) trials, congruent Flanker and incongruent SRC (FcSi), incongruent Flanker and SRC (FiSi) trials, and neutral trials (see Figure 1).

Reaction times (RT) of correct trials and error rates (ERs) for both groups are depicted in Figures 2A,B. RTs and ERs were entered into a 2 × 2 × 2 repeated measures ANOVA with the within-group factors Flanker and SRC and Age as a between-group factor. There was a significant main effect for all factors with regard to RTs [Flanker: *F*_(1, 37)_ = 109.368, *p* < 0.001; SRC: *F*_(1, 37)_ = 49.202, *p* < 0.001; Age: *F*_(1, 37)_ = 21.478, *p* < 0.001; see Table 1]. In comparison to congruent trials, RTs were slower in incongruent trials with regard to the Flanker conflict (congruent: *M* = 575 ms, s.e.m. = 14; incongruent: *M* = 612 ms, s.e.m. = 15) and the SRC (congruent: *M* = 577 ms, s.e.m. = 14; incongruent: *M* = 610 ms, s.e.m. = 16) task. Elderly participants presented larger RTs than young controls (young: *M* = 527, s.e.m. = 20; old: *M* = 660, s.e.m. = 21), and the interaction of SRC and Age became significant [*F*_(1, 37)_ = 6.000, *p* = 0.019]. Both groups showed larger RTs for the incongruent condition of the SRC task, but the SRC congruency effect was significantly larger in elderly (Δ RT = 45 ms, s.e.m. = 36) compared to young participants [Δ*RT* = 22 ms, s.e.m. = 22; *t*_(37)_ = −2.45, *p* = 0.019; see Figure 2]. There was no Flanker × Group interaction [*F*_(1, 37)_ = 1.162, *p* = 0.288] or a Flanker × SRC interaction [*F*_(1, 37)_ = 0.20, *p* = 0.890].

**Table 1:** **Statistical values of the behavioral data analysis (RT: reaction times; ER: error rates, SRC: stimulus response conflict)**.

	**RT**		**ER**	
	***F*-value**	***p*-value**	***F*-value**	***p*-value**
Flanker	109.37	<0.001	15.66	<0.001
SRC	49.20	<0.001	16.23	<0.001
Age	21.48	<0.001	0.52	0.821
Flanker x Age	1.16	0.288	4.80	0.035
SRC x Age	6.00	0.019	0.57	0.455
Flanker x SRC	0.20	0.890	3.05	0.089
Flanker x SRC x Age	0.32	0.572	1.17	0.286

For ERs a significant main effect was found for the factors Flanker [*F*_(1, 37)_ = 15.667, *p* < 0.001] and SRC [*F*_(1, 37)_ = 16.226, *p* < 0.001], but not for the factor Age [*F*_(1, 37)_ = 0.052, *p* = 0.821]. The incongruent condition (*M* = 6.2, s.e.m. = 1.2) of the SRC elicited higher ERs as compared to the congruent condition (*M* = 2.1, s.e.m. = 0.31). The significant main effect for the factor Flanker resulted from higher ERs in incongruent (*M* = 5.1, s.e.m. = 0.74) compared to congruent Flanker trials (*M* = 3.2, s.e.m. = 0.75). However, the interaction of Flanker × Age became significant [*F*_(1, 37)_ = 4.804, *p* = 0.035]. The difference between incongruent and congruent Flanker trials was significantly higher in young participants (Δ*ER* = 3.1, s.e.m. = 0.72) in comparison to elderly [Δ*ER* = 0.88, s.e.m. = 0.68; *t*_(37)_ = 2.19, *p* = 0.035]. There was no significant interaction of SRC × Age [*F*_(1, 37)_ = 0.57, *p* = 0.455].

Taken together, the behavioral data analysis revealed significant effects for both conflict types in both groups with regard to RTs. The congruency effect of the SRC task was significantly larger in elderly participants. Additionally, ERs were modulated by the SRC task in both groups. The Flanker congruency effect for ERs was only evident in young adults.

### Imaging data

In a first approach the analysis of imaging data was based on separately comparing incongruent Flanker [FiSc], incongruent SRC [FcSi], and the double incongruence [FiSi] condition against the double congruence condition [FcSc]. All contrasts were calculated for each group and entered into a between group analysis.

**Figure 3:**
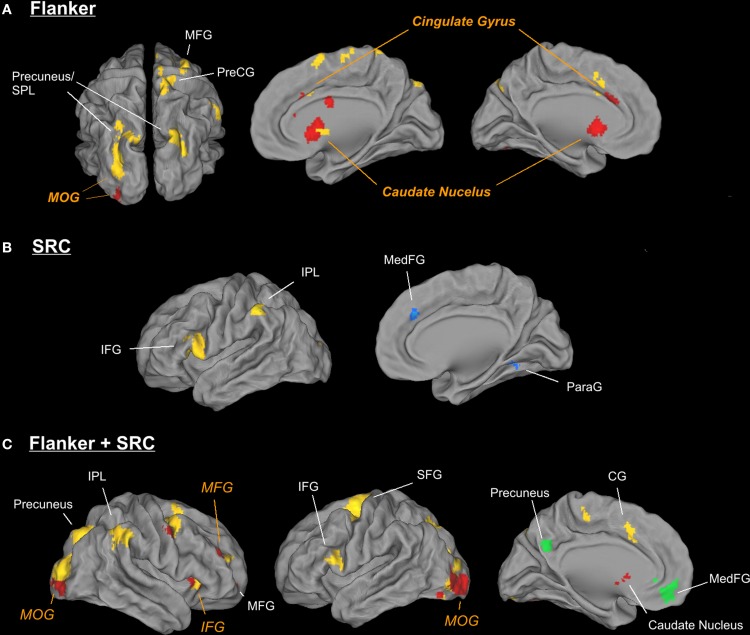
**Illustration of different activation patterns for the Flanker (A), the SRC (B), and the double conflict (C) conditions in young and elderly participants**. Activation sites of elderly participants are displayed in yellow (activation) and green (deactivation), while active regions of young participants are displayed in red (activation) and blue (deactivation). For the purpose of illustration the threshold was set to *p* < 0.005 (uncorrected) and *k* = 50.

In young participants the Flanker conflict resolution ([FiSc] > [FcSc]) revealed bilateral activation in the caudate nucleus (Cd), in the anterior cingulate gyrus (ACC) and the middle occipital gyrus (MOG) in the right hemisphere. The same contrast in the elderly group elicited signal increases in bilateral occipito-parietal areas including the precuneus (bilateral), the left MOG, the right superior parietal lobule (SPL), the cingulate gyrus, the MFG and the pre-central gyrus (see Table 2 and Figure 3A).

**Table 2:** **Peak activations for the Flanker [FiSc > FcSc], SRC [FcSi > FcSc], and double conflict (Flanker + SRC) [fisi > fcsc] contrasts, for younger and elderly participants**.

	**Young**					**Old**				
	***x*,**	***y*,**	***z***	***t*-value**	**Cluster-size**	***x*,**	***y*,**	***z***	***t*-value**	**Cluster-size**
**FLANKER CONFLICT**										
Cingulate gyrus	6,	4,	29	4.24	12	−2,	16,	40	4.29	97
Middle frontal gyrus						34,	4,	61	3.95	10
Precentral gyrus						16,	−32,	72	4.21	27
Precuneus						26,	−76,	42	4.86	89
						−24,	−80,	24	4.71	56
Middle occipital gyrus	−36,	−72	−13	4.32	31	−26,	−92,	16	3.80	10
Caudate	−6,	12,	0	5.25	138					
	−12,	28,	0	4.08	11					
**SRC**										
Inferior frontal gyrus						−54,	18,	8	4.33	22
Medial frontal gyrus	10,	36,	32	−4.11	9					
Inferior parietal lobule						−58,	−36,	48	6.05	35
Superior temporal gyrus	48,	−38,	18	−4.291	12			
Parahippocampal gyrus	18,	−48	−11	−4.45	14					
**FLANKER CONFLICT + SRC**										
Inferior frontal gyrus	34,	34,	−8	4.34	24	−58,	16,	16	4.33	25
Middle frontal gyrus	30,	52,	−11	3.95	11	30,	6,	56	4.82	127
Middle frontal gyrus	56,	6,	42	4.46	15	44,	8,	45	4.53	30
Superior frontal gyrus						−16,	14,	45	6.20	308
Medial frontal gyrus						−2,	14,	48	3.86	16
Medial frontal gyrus						2,	−26,	56	3.95	22
Medial frontal gyrus						2,	50,	−16	−4.84	112
Anterior cingulate	12,	22,	−8	4.16	16					
Precuneus						18,	−80,	40	4.50	93
Precuneus						−20,	−68,	48	4.47	24
Precuneus						−6,	−60,	32	−4.30	15
Inferior parietal lobule						66,	−30,	37	4.11	27
Post−central gyrus	32,	−38,	72	4.25	11					
Middle temporal gyrus						−44,	−2	−37	−4.56	15
Fusiform gyrus						−18,	−90	−19	3.96	10
Middle occipital gyrus	36,	−92	−5	5.4885	67	30,	−88	−3	5.23	260
Middle occipital gyrus	−36,	−74	−16	4.30	15	−30,	−96,	16	4.18	44
Inferior occipital gyrus	−30,	−98	−8	4.91	157					
Caudate	6,	12,	8	4.13	23					

All reported regions are significant at p < 0.001 (uncorrected) with a spatial extent threshold of k = 9. Abbreviations: BA, Brodmann area; x, y, z = MNI coordinates of peak activations.

The data of the conjunction analysis (Table 3) showed a bilateral activation pattern in the head of Cd during Flanker conflict processing, and group related differences in Pre-CG and in MOG. While in Pre-CG Flanker related signal increase was more pronounced in elderly participants, in MOG only young participants showed stronger activation in response to the Flanker conflict.

**Table 3:** **Peak activations derived from a conjunction analysis [intermediate null hypothesis, *p* < 0.005 (uncorrected), *k* = 9] and an interaction analysis (*p* < 0.001, uncorrected, *k* = 9) of the Flanker [fisc > fcsc], SRC [fcsi > fcsc], and double conflict (Flanker + SRC) [fisi > fcsc] contrasts**.

	**Conjunction**					**Interaction**				
	***x*,**	***y*,**	***z***	***t*-value**	**Cluster-size**	***x*,**	***y*,**	***z***	***t*-value**	**Cluster-size**
**FLANKER CONFLICT**										
						**Old > Young**				
Precentral gyrus						18,	−34,	72	3.85	12
						**Young > Old**				
Middle occipital gyrus						−36,	−74	−11	3.79	17
Caudate	8,	12,	5	3.28	57					
	−6,	12,	0	3.00	12					
						**Old > Young**				
**SRC**										
Inferior frontal gyrus						−44,	12,	21	5.04	60
Inferior parietal lobule						−58,	−34,	48	4.17	21
**FLANKER CONFLICT + SRC**										
Inferior frontal gyrus	34,	28,	−3	3.13	23					
Medial frontal gyrus						−18,	12,	43	3.99	12
Precuneus	26,	−72,	37	3.11	10					
Middle occipital gyrus	−36,	−90,	0	3.79	87					
	34,	−92	−3	4.69	145					

*In addition, group contrasts thresholded at p < 0.005 for young and elderly participants respectively were used as an inclusive mask. Abbreviations: BA, Brodmann area; x, y, z = MNI coordinates of peak activations*.

The analysis of the SRC conflict resolution ([FcSi] > [FcSc]) revealed no significant signal change in the young group. We found various activations for the reverse contrast ([FcSc] > [FcSi]) including the parahippocampal gyrus (ParaG), the IPL, and the medial frontal gyrus (MedFG,). In elderly participants we observed a significant signal increase in the IFG and the IPL (see Figure 3B). There were no significant results in the conjunction analysis of both groups, but a significant interaction in IFG and IPL. Here, SRC related activation only became evident in the elderly subject group.

In the double conflict condition (i.e., trials containing the SRC and the Flanker conflict, [FiSi] > [FcSc]) we found a significant activation comprising a frontal cluster (IFG, MFG and MedFG), posterior (MOG and IOG, Post-CG), and subcortical areas (Cd; see Figure 3C and Table 3) in young participants. In elderly individuals double conflict trials were associated with a signal increase in IFG, MFG, SFG and the ACC. In addition, we found a significant activation in the precuneus, the IPL, the MOG and the fusiform gyrus (FG). Deactivations in elderly individuals were found in MedFG, precuneus and middle temporal gyrus. The conjunction analysis for the double conflict condition revealed a common activation cluster in the MOG, the right IFG and the precuneus. The interaction analysis between both groups showed a significant activation in SFG for elderly participants only.

## Discussion

The aim of the present study was to investigate whether and how ageing differentially affects interference control using different types of conflicts: a Flanker and a SRC conflict as well as a combination of both. The analysis of the behavioral data showed that both young and elderly participants presented Flanker conflict and SRC related effects. RTs were modulated by the Flanker conflict in both groups. Young as well as elderly participants responded significantly faster in congruent compared to incongruent Flanker trials. Error rates in the Flanker task, however, were only increased in young participants. These data correspond to previous studies demonstrating that the Flanker effect does not increase with higher age, and higher accuracy rates in older adults are repeatedly reported in studies using the Flanker task (Hsieh and Fang, 2008; Wild-Wall et al., 2008). These findings may be attributable to different strategies in Flanker task processing. For example, elderly might use a more conservative decision criterion, probably causing to prioritize correct over fast responses (Hsieh and Fang, 2008). For the SRC task performance both groups showed significantly shorter response latencies in the congruent in comparison to the incongruent SRC condition. This effect was larger in elderly than in young participants, and corresponds with various reports in the literature (van der Lubbe and Verleger, 2002; Kubo-Kawai and Kawai, 2009; Kawai et al., 2012).

### Flanker task

The behavioral findings are corroborated by the analysis of the fMRI data which showed that interference resolution in different conflict types rely on distinct neural networks and that ageing has differential effects on conflict processing and their neural correlates. In young adults neuronal activation induced by the Flanker conflict was found in a network comprising MOG, ACC, and Cd. In elderly participants, Flanker incongruent trials elicited activation in MOG, precuneus, cingulate gyrus, MFG and Pre-CG. Although both groups showed increased activation in MOG and cingulate gyrus there was no direct overlap of the activated clusters. A conjunction analysis revealed common bilateral activation in the Cd. However, the caudate activation in the group contrast of the elderly participants only became evident when lowering the threshold to *p* < 0.002. These findings indicate that the older and younger participants partly share similar mechanisms of Flanker conflict processing. Comparable activation patterns in young and elderly individuals during conflict resolution have also been reported in previous studies introducing the Stroop and the Flanker tasks (Langenecker et al., 2004; Zysset et al., 2007; Zhu et al., 2010). These data suggest a “core network” comprising cingulate cortex and basal ganglia activation underlying cognitive control processes in the respective subgroups. Within the framework of conflict processing theories the cingulate cortex is associated with evaluative functions such as conflict detection and task monitoring (Carter et al., 1998; Kiehl et al., 2000; van Veen and Carter, 2002) and/or response selection (Liu et al., 2004). The basal ganglia also seem to play a role in cognitive control processing. Besides clinical data that showed impaired decision making, task switching and flexibility in patients with basal ganglia dysfunctions (e.g., Parkinson's Brown and Marsden, 1990; Cools et al., 2001; Brand et al., 2005; Mimura et al., 2006 or Huntington's disease Hanes et al., 1995; Aron et al., 2003b) there is also evidence from neuroimaging studies including healthy participants demonstrating that the basal ganglia are also consistently found to be involved in tasks, which demand high cognitive control during decision making (Tanaka et al., 2004; Forstmann et al., 2008). In particular, the Cd seems to play a critical role in adjusting the decision criteria (Forstmann et al., 2008; Kuchinke et al., 2011). Thus, the Cd activation in resolving incongruent Flanker trials might reflect the participants' adjustment of the response thresholds in order to avoid response activation triggered by the irrelevant input channel.

Besides the aforementioned common activation pattern, there were also significant differences between groups. An interaction analysis revealed that elderly showed additional activation in Pre-CG, while young participants additionally recruited a posterior cluster comprising IOG and MOG. In addition, within-group comparisons showed an increased MFG and precuneus activation in elderly participants only. This recruitment of additional brain areas in elderly participants was also shown in previous studies of conflict control (Langenecker et al., 2004; Zysset et al., 2007). We suppose this additional fronto-parietal activation patterns in older participants to reflect compensatory control mechanisms such as increased efforts in spatial attention and top-down attentional control or additionally updating task set information to optimize performance (e.g., the precuneus activation, see Jahn et al., 2011).

### SRC task

In contrast to the Flanker conflict incongruent SRC trials were mainly associated with a signal decrease in the IPL, the MedFG, and the ParaG in young participants. activation similar pattern in young participants during the processing of incongruent SRC trials was also found by Lee et al. (2006). Furthermore, a previous study from our own group using a similar double conflict task design (Fruhholz et al., 2011) also yielded deactivations in incongruent SRC trials in young adults. These data may indicate that young participants benefit from corresponding spatial information as found in double congruence trials and thus might rather reflect a facilitation than an interference effect. This hypothesis is corroborated by previous studies showing medial frontal areas involved in facilitatory effects in conflict tasks. Swick and Jovanovic (2002) presented a case study in which a patient with a lesion in the mid caudal area of the ACC showed a lack of facilitation in a Stroop task when compared to healthy controls. Furthermore, activation in several regions of the medial frontal wall was also demonstrated during congruent Stroop trials in a positron emission tomographic study with healthy adults (Carter et al., 1995). Further active clusters in the ParaG and the IPL might represent an enhanced processing of visual characteristics (shape) of the arrows that are transferred into spatial cues (Bar et al., 2001; Spiridon and Kanwisher, 2002). On the other hand, elderly participants showed increased activation in IFG and IPL during incongruent SRC trials. The IFG plays a substantial role in implementing inhibitory control and is frequently recruited during the processing of conflict tasks (Aron et al., 2003a; Chikazoe et al., 2007; Hampshire et al., 2010). In addition, age-dependent activity increase was found in the IFG in a Stroop task (Langenecker et al., 2004; Zysset et al., 2007). The IPL is usually associated with processing of visuo-spatial information and attentional top-down control. Thus, the fronto-parietal activation patterns in elderly participants might reflect additional demands of inhibitory control and attentional resources to resolve the SRC task.

In contrast to the Flanker task, there were no common activation patterns in young and elderly adults while resolving the SRC task. This finding may indicate that both groups make use of different strategies when irrelevant information interferes with correct response selection. While younger participants seem to also consider irrelevant information for response selection, elderly participants show an activation pattern that is typically associated with inhibition control. The present finding of neuronal activation in the SRC task does not confirm studies of interference control in ageing that found similar neural substrates of inhibition in both age groups (Langenecker et al., 2004; Zysset et al., 2007; Zhu et al., 2010). The present behavioral and imaging data rather indicate that SRC is processed qualitatively different in elderly and younger individuals.

### Double conflict condition

In young participants, trials demanding the simultaneous resolution of two different types of conflicts elicited activation of a network comprising MOG, Cd, IFG, and MFG. This pattern shares similarities with the activation found in the single Flanker condition, but extends to the ventrolateral prefrontal (BA 47) and the orbitofrontal cortex (BA 11). This finding corroborates the fMRI data of previous studies of our group where we introduced double conflict paradigms in young adults. Although the type of conflicts differed between studies we found a distinctive activation pattern for the double conflict conditions in the MFG (Wittfoth et al., 2009) and in the frontopolar cortex (Fruhholz et al., 2011). Thus, the additional recruitment of middle and inferior frontal areas during monitoring of simultaneously presented conflict types in the present study might indicate the implementation of superordinate control mechanisms that regulate the processing of higher conflict situations. Elderly participants showed stronger activations and a more extended neural network during double conflict trials comprising MOG, precuneus, MFG, and ACC (comparable to the single Flanker condition) and additional activations in the left IPL and the IFG (as also found in the single SRC condition). The conjunction analysis demonstrated a network of brain areas, which is recruited by both groups during double conflict trials comprising bilateral MOG and right IFG and precuneus. The additional frontal medial wall activity in elderly participants as demonstrated by an interaction analysis (Table 2) might be attributable to the generally higher task demand of double conflict trials due to the higher amount of interference.

In conclusion, these findings suggest that elderly and young participants use similar networks to resolve interference from two concurrent conflicts, which however seem to be more pronounced and leading to an additional recruitment of MedFG in the elderly.

### Ageing effects on different conflict types

Taken together, these data corroborate the hypothesis that different components of interference control evolve differently with advanced age. While the core neural mechanisms underlying S-S conflict resolution remain stable, neural processing of S-R conflicts is substantially altered in elder participants. Turner and Spreng (2012) demonstrated that different types of executive functions, such as working memory and inhibition, show distinctive neural age-related effects. Though they provide no information about how different components of interference control develop with increasing age, this review clearly demonstrates that ageing does not have a unitary effect on different executive functions. Another recent study (Sebastian et al., 2013) comprising a Go/Nogo, a Simon and a Stop Signal task confirmed that differential ageing effects are also found in different components of inhibition. While the Go/NoGo and Simon tasks showed an increased neural activation in prefrontal areas in elderly participants, a decrease of neural activity was found during the Stop Signal task in the same group. Our findings are also in line with the study of Kawai et al. (2012) analyzing the neural processing of Flanker and Simon conflicts in young and elderly participants using NIRS. In this study, the pattern of age-dependent neural effects differed between both conflict tasks. Elderly participants showed increased bilateral activation in the SFG in the Simon task, while Flanker task was associated with increased activation in the right MFG and SFG.

Several studies clearly demonstrate that ageing goes along with deficiencies in the inhibition of distracters, while neural mechanisms related to the processing of the target stimulus largely remain intact (West and Alain, 2000; Fockert et al., 2009; Wascher et al., 2012). As both the S-S and S-R conflicts require the inhibition of distracting information, the inhibition of SRC task-related distracters is particularly vulnerable to age-related alterations. Wild-Wall et al. (2008) reported electrophysiological markers reflecting the processing of the target stimulus in a Flanker task to be enhanced in elderly participants. They also found lower error rates in elderly compared to young participants and argued that elderly participants show a stronger attentional focus on the target stimulus to reduce interference induced by the Flanker stimuli. A similar strategy might have also been employed by the elderly participants in the present study. The smaller Flanker effect in the error rates of elderly participants supports the hypothesis that elderly focus on the target stimulus and thus lower the processing of flanking distracters. Furthermore, additional activation of frontal and parietal areas in elderly in the incongruent Flanker condition might indicate additional top-down regulation that amplifies relevant (target) and attenuates irrelevant (flanker) input channels. However, this strategy does not promote interference reduction for SRC-related distracters since they are confounded with the target stimulus (Proctor et al., 2005). In contrast, an enhanced processing of the target stimulus might lead to a concomitant deeper processing of SRC-related distracters and thus stronger SRC induced interference. Elderly participants presumably do not employ a compensatory strategy to reduce interference resulting from SRC distracters. Thus, the recruitment of IFG and IPL seem to reflect these additional demands for conflict resolution. In conclusion, the present data demonstrate that ageing generally does not impair conflict resolution, but differentially interferes with different subcomponents of conflict processing.

## Author contributions

Conception of work: Margarethe Korsch, Sascha Frühholz, Manfred Herrmann; data acquisition: Margarethe Korsch; data analysis and interpretation: Margarethe Korsch, Manfred Herrmann; draft of work: Margarethe Korsch; critical revision: Margarethe Korsch, Sascha Frühholz, Manfred Herrmann. All authors are in agreement to be accountable for all aspects of the work in ensuring that questions related to the accuracy or integrity of any part of the work are appropriately investigated and resolved.

### Conflict of interest statement

The authors declare that the research was conducted in the absence of any commercial or financial relationships that could be construed as a potential conflict of interest.
